# Antidiabetic Activity of Potential Probiotics *Limosilactobacillus* spp., *Levilactobacillus* spp., and *Lacticaseibacillus* spp. Isolated from Fermented Sugarcane Juice: A Comprehensive In Vitro and In Silico Study

**DOI:** 10.3390/nu15081882

**Published:** 2023-04-13

**Authors:** Chandana Kumari V. B., Sujay S. Huligere, Ghallab Alotaibi, Abdulaziz K. Al Mouslem, Ammar Abdulraheem Bahauddin, Thippeswamy Boreddy Shivanandappa, Ramith Ramu

**Affiliations:** 1 Department of Biotechnology and Bioinformatics, JSS Academy of Higher Education and Research, Mysuru 570015, Karnataka, India; chandanavb2@gmail.com (C.K.V.B.); sujayhuligere@gmail.com (S.S.H.); 2 Department of Pharmaceutical Sciences, College of Pharmacy, Shaqra University, Al-Dawadmi Campus, Shaqra 11961, Saudi Arabia; ghalotaibi@su.edu.sa; 3 Department of Pharmaceutical Sciences, College of Clinical Pharmacy, King Faisal University, Al Ahsa 31982, Saudi Arabia; aalmoslem@kfu.edu.sa; 4 Department of Pharmacology and Toxicology, College of Pharmacy, Taibah University, Madinah 42535, Saudi Arabia; dr.ammar.b@hotmail.com; 5 Department of Biomedical Science, College of Pharmacy, Shaqra University, Al-Dawadmi Campus, Shaqra 11961, Saudi Arabia; t_swamy@hotmail.com

**Keywords:** sugarcane, α-glucosidase, α-amylase, probiotic, lactic acid bacteria

## Abstract

Probiotics are regarded as a potential source of functional foods for improving the microbiota in human gut. When consumed, these bacteria can control the metabolism of biomolecules, which has numerous positive effects on health. Our objective was to identify a probiotic putative *Lactobacillus* spp. from fermented sugarcane juice that can prevent α-glucosidase and α-amylase from hydrolyzing carbohydrates. Isolates from fermented sugarcane juice were subjected to biochemical, molecular characterization (16S rRNA) and assessed for probiotic traits. Cell-free supernatant (CS) and extract (CE) and also intact cells (IC) were examined for the inhibitory effect on α-glucosidase and α-amylase. CS of the strain showed the highest inhibition and was subjected to a liquid chromatography–mass spectrometry (LCMS) analysis to determine the organic acid profile. The in silico approach was employed to assess organic acid stability and comprehend enzyme inhibitors’ impact. Nine isolates were retained for further investigation based on the preliminary biochemical evaluation. *Limosilactobacillus* spp., *Levilactobacillus* spp., and *Lacticaseibacillus* spp. were identified based on similarity > 95% in homology search (NCBI database). The strains had a higher survival rate (>98%) than gastric and intestinal fluids, also a high capacity for adhesion (hydrophobicity > 56%; aggregation > 80%; HT-29 cells > 54%; buccal epithelial cells > 54%). The hemolytic assay indicated that the isolates could be considered safe. The isolates’ derivatives inhibited enzymes to varying degrees, with α-glucosidase inhibition ranging from 21 to 85% and α-amylase inhibition from 18 to 75%, respectively. The CS of RAMULAB54 was profiled for organic acid that showed the abundance of hydroxycitric acid, citric acid, and lactic acid indicating their role in the observed inhibitory effects. The in silico approach has led us to understand that hydroxycitric acid has the ability to inhibit both the enzymes (α-glucosidase and α-amylase) effectively. Inhibiting these enzymes helps moderate postprandial hyperglycemia and regulates blood glucose levels. Due to their promising antidiabetic potential, these isolates can be used to enhance intestinal health.

## 1. Introduction

Over the past several decades, there has been a dramatic rise in the prevalence of type 2 diabetes mellitus, which is defined by unusually high blood glucose levels followed by a relative insulin insufficiency [1]. According to certain suggestions that the development of noticeable hyperglycemia is preceded by insulin resistance. Insulin resistance in the liver leads to an ineffective reduction of glucose production as a result of hyperglycemia and glucose intolerance [2]. Increased incidences of diabetes with high morbidity and death rates are a result of food and lifestyle changes [3]. The side effects of diabetic drugs open the door to investigating conventional or alternative treatments. Future developments in personalized nutrition and probiotic approaches, also the recognition of therapeutic probiotic constituents, and the use of genetically modified bacteria that express therapeutic factors in microbiota are some potential gastrointestinal-based strategies to lower glucose levels [4,5]. The primary and most promising targets of pharmaceutical intervention to decrease hyperglycemia are enzymes such as α-glucosidase and α-amylase [6]. The complex oligo/di saccharides are broken down by several enzymes, such as α-amylase and α-glucosidase, and then transformed into monosaccharide, which is absorbed in the intestine [7,8].

Numerous studies relate changes in the prosperity of the gut microbiota and the onset of diabetes. Additionally, the gut microbiota, one of the most crucial elements in the regulation of host health, is mostly found in the intestine [9,10,11]. The adult human gut is home to roughly ten times as many cells as there are in the rest of the human body [7,12]. Various researchers have identified lactic acid bacteria (LAB), which may substantially impact diabetes symptoms [13,14,15,16,17]. Many researchers have recovered LAB from several sources, including carrot human breast milk, beetroot juice, newborn feces, batters, and fermented foods [10,18,19,20].

Recent investigations have also demonstrated the presence of LAB in factories that process sugarcane and in spills of sugarcane [21,22]. A perennial grass of the Poaceae family known as sugarcane, or *Saccharum officinarum* Linn., is mostly grown for its juice, which is used to make sugar. The majority of the sugarcane in the world is grown in tropical and subtropical regions. They chew raw sugarcane to extract the juice, utilize syrup as a sweetener, and also drink fermented sugarcane juice [23]. In areas where the plants are grown, sugarcane parts are frequently used as animal feed. Ruminants use the leaves as a source of fodder, and studies have shown positive effects on milk production, intake, digestion, fatty acid composition, chewing activity, and ruminal fermentation when the wheat straw is substituted with sugarcane bagasse in mid-lactation dairy cow diets [24]. Researchers employed *Lactobacillus* spp. to improve the quality of sugarcane bagasse used as ruminant feed. They also obtained phenolic flavoring substances [25,26]. *Leuconostoc* spp. and *Lactobacillus* spp. recovered from the sugarcane processing stream [27]. Some studies emphasize the aid of *Lactobacillus* spp. for improving fermentation of the silage, bagasse, and profiling species obtained in the sugarcane juice and sugarcane processing steams [28,29,30]. Ellis et al. claim that filtered sugarcane molasses concentrate, which is high in minerals and phytonutrients and is typically ingested with foods that include carbohydrates as a functional ingredient, has the capacity to reduce insulin responses and lessen the load on the pancreatic beta cells. The sugarcane extracts with high polyphenol content may aid in preventing glucose and fructose absorption by intestinal cells and reactivating insulin synthesis in damaged β-cells, both of which are crucial for controlling diabetic symptoms [31]. Organic chromium (chromium III) in the diet has a role in glucose tolerance. White sugar contains chromium levels that are 35 times lower than those of sugarcane juice; it appears that consuming sugarcane juice for a month will boost chromium levels. In their preliminary investigation, Ayuningtyas et al. showed the potential of sugarcane juice to replace white sugar in the treatment of diabetes mellitus [32]. According to a study by Abduldileep et al., potential sucrase inhibitors of sugarcane may block mammalian sucrases. Human sucrase and rat intestine α-glucosidases have been demonstrated to be more selectively inhibited by the proteinaceous invertase protein sucinh (inhibitor of sugarcane and analogous to maize) than by isomaltase and human maltase-glucoamylase [33]. This is the first study that uses fermented sugarcane juice to identify LAB with potential antidiabetic and probiotic properties. From this perspective, the major objective of the work was to isolate LAB from fermented sugarcane juice as a source with putative probiotic characteristics and the potential to hinder the enzymes that hydrolyze carbohydrates, specifically α-glucosidase and α-amylase.

## 2. Materials and Methods

### 2.1. Sugarcane Juice Fermentation, Isolation, and Preliminary Biochemical Characterization

Sugarcane purchased from the local market (Mysuru, Karnataka) was cleaned and rinsed with water. The hard outer covering was peeled off with a sharp, sterilized knife, and was cut and sliced into small pieces. The chopped pieces were ground to extract the juice and the strainer was used to filter the tiny particles. The extracted juice was subjected to fermentation period for 24 h room-temperature (25 °C). *Lactobacillus* MRS agar (*Lactobacillus* DeMan, Rogosa, and Sharpe agar, HiMedia Laboratories Pvt. Limited, Mumbai, India) plates were used to isolate (serial dilution) the colonies. The colonies were screened for Gram’s staining and catalase tests. The cell preparation, preliminary and biochemical assays (tolerance to temperature, pH, NaCl, and carbohydrates fermentation) were performed as methodology mentioned by Kumari et al. with slight modification [34].

### 2.2. Fermented Sugarcane Juice Strains Molecular Identification and Phylogenetic Analysis

The isolates were subjected to DNA isolation and amplification based on their probiotic potential. The universal primers 27-F and 1492-R, as reported by Kumari et al., were employed with a few modifications to amplify the 16S rRNA sequencing of the isolated LAB isolates [34]. After sequencing the polymerase chain reaction (PCR) amplified product was subjected to BLAST (basic local alignment search tool). The sequences received accession numbers after being added to the GenBank database [34]. MEGA X was used to create the phylogenetic tree for the nine sequences of the LAB isolates from the current investigation (Version 10.2.4). Likelihood phylogenetic trees were created with a 100 bootstrap consensus tree. The most accurate model was Tamura-Nei [35]. An initial tree (or trees) for the heuristic search was automatically created by using the Neighbor-Join and BioNJ algorithms on a matrix of pairwise distances [36].

### 2.3. Probiotic Properties

#### 2.3.1. Assessment of Adhesion Capability

##### Cell Surface Hydrophobicity, Autoaggregation, and Coaggregation Assay

The isolate’s cell surface hydrophobicity, autoaggregation, and coaggregation were evaluated using the method described by Tuo et al. with a minor modification [37]. The hydrophobicity and aggregation of the cell surface were calculated using the following formula:

Aggregation (%) = [(Xo − X)/Xo] × 100

Initial absorbance (600 nm) is denoted by “Xo” in the equation, and final absorbance is denoted by “X”.

For the coaggregation assay, 4 mL of the pathogenic strains *B. subtilis*, *E. coli*, *P. aeruginosa*, *M. luteus*, and *S. typhimurium* were combined with a 2 mL suspension of the LAB isolates (1 × 10^8^ CFU/mL) and incubated at 37 °C, 2 h. At 600 nm, the mixture’s absorbance was determined. As shown below, the coaggregation (%) is calculated:

Coaggregation (%) = [(X_LAB_ + Y_P_) − Z_mix_]/(X_LAB_ + Y_P_) × 100

where, Z_mix_ = the pathogen-LAB combination’s absorbance at time 2 h and (X_LAB_ + Y_P_) depicts the mixture’s absorbance at time 0 h, respectively.

##### Human Buccal Epithelial Cells, and HT-29 Cells In Vitro Adhesion Assay

Using Kumari et al. methodology, the isolate’s in vitro adhesion properties to buccal epithelial cells and the human colorectal cancer cell line (HT-29 cells) were evaluated [38]. LAB adherence to buccal epithelial cells was determined through microscopic inspection using Gram’s staining. A 70% confluent HT-29 cell plate was treated (60 min; 37 °C) with 1 mL of bacterial suspension (10^8^ CFU/mL) to measure the isolate’s adhesion potential to the cells (5% CO_2_ atmosphere). After removing non-adherent bacterial cells with PBS, serial dilution was carried out before being plated (37 °C, 24 h). Using the CFU/mL ratio between the initial number of bacteria sown and the amount of bacteria seeded after washing, the ability of the bacteria to adhere to the cells was evaluated. Each experiment was conducted three times in pairs.

#### 2.3.2. Tolerance Assay

##### Tolerance to Bile Salt in Acidic Conditions

The bile salt tolerance in an acidic environment was evaluated, with a few minor modifications, following Begley et al. [39]. For 0, 2, and 4 h, isolated LAB strains (10^8^ CFU/mL) were introduced to 0.3 and 1% ox gall MRS broth (pH 2, 37 °C). After a 24 h incubation period (37 °C), the MRS agar plate was used for enumeration. The viability rate was calculated using the formula below (%):

Viability (%) = (X/X_0_) × 100

where, X- Log CFU/mL of LAB (viable) at time (2 and 4 h) and X_0_- Log CFU/mL of LAB at time (0 h).

##### Assay for Simulated Gastric Juice Tolerance

Pepsin (3000 mg/L of PBS; pH 3, 2500 U/mg, Sisco Research Laboratory Pvt. Ltd., Mumbai, India) and trypsin (1000 mg/L of PBS; pH 8, 2000 U/g, Sisco Research Laboratory Pvt. Ltd.) were dissolved and sterilized (0.22 μm/L) to establish simulated gastric juice and intestinal juice conditions, respectively. The isolates’ ability to withstand the digestion process under gastric and intestinal conditions for up to 3 h and 8 h, respectively, was assessed. The designated strain’s gastrointestinal tolerance was evaluated using viable colony counts [40]. The survival rate was calculated using the formula shown below:

Survival rate (%) = [(N_1_/N_0_)] × 100

where, N_1_ = After treated with simulated gastrointestinal fluids, the total viable count of LAB strains, and N_0_ = Before treatment, the total number of viable LAB strains [41].

### 2.4. Antibacterial Activity

Using the agar well diffusion method, the isolate’s antibacterial activity against pathogenic bacteria was determined [42]. Bacillus cereus (MTCC-1272), Bacillus subtilis (MTCC1-0403), Escherichia coli (MTCC-443), Klebsiella aerogenes (MTCC-2822), Klebsiella pneumonia (MTCC-10309), Micrococcus luteus (MTCC-1809), Pseudomonas aeruginosa (MTCC-424), Pseudomonas fluorescens (MTCC-667), Salmonella typhimurium (MTCC-98), and Staphylococcus aureus (MTCC-1144) were the test organisms. The 100 µL test organisms were added to LB agar (Luria Bertani agar) plates in a uniform distribution. Wells were bored into the plates using borers. 100 µL of LAB isolates that had been cultivated overnight were placed in each well.

### 2.5. Antibiotic Susceptibility

On MRS agar plates, the isolate’s antibiotic susceptibilities were examined using the antibiotic disc diffusion technique. LAB isolates (10^8^ CFU/mL) were transferred to the MRS agar plates and allowed to dry. The plates were then loaded with antibiotic discs (37 °C; 24 h). Using 100 µg/discs of streptomycin, 30 µg/discs of vancomycin, and tetracycline, 15 µg/discs of azithromycin, 10 µg/discs of methicillin and ampicillin, the antibiotic susceptibility pattern of the isolates was identified. The results of antimicrobial disc susceptibility tests were classed as sensitive, moderately susceptible, or resistant based on the interpretation zone diameters and performance characteristics [43].

### 2.6. Hemolytic Activity

Husain et al. [44] described the method, which was applied to evaluate the isolate’s hemolytic activity with a few minor methodological modifications. Following streak plate inoculation, the isolates were cultured (48 h; 37 °C) on blood agar plates (5% sheep blood). Red blood cells were lysed in the media surrounding the colonies to determine the isolates’ hemolytic activity (α, β, γ -hemolysis) [44].

### 2.7. Screening for Antioxidant Activity

The isolates’ ability to scavenge ABTS radicals was examined using the approach described by Silva-Rivas et al., with a few minor modifications [45]. Furthermore, the DPPH radical-scavenging capability was carried out in accordance with the earlier research by Li et al. with slight modification [42].

### 2.8. Inhibitory Assay for Carbohydrate Hydrolyzing Enzymes

The protocol indicated by Kumari et al. was followed in the preparation of the cells [38]. The inhibition was carried out using intact cells (IC), cell-free supernatant (CS), and cell-free extract (CE). Shimojo et al.’s method for inhibiting α-glucosidase was followed, but with minor alterations [46]. According to Talamond et al.’s instructions, the experiment on α-amylase inhibition was carried out [47]. In diabetics, inhibiting the digestive enzymes intestinal α-glucosidase and pancreatic α-amylase, which are in function breaking down and absorbing carbohydrates, may reduce postprandial hyperglycemia [6,10,46,48]. Using a microplate reader (Multiskan FC, ThermoFisher Scientific, Mumbai, India), the absorbance of the reaction mixture and was determined at 405 nm for α-glucosidase and 540 nm for α-amylase.

Inhibition (%) = (1 − X_S_/X_C_) × 100 (1)

where X_S_ = absorbance of the reactants with the sample; X_C_ = absorbance of the reactant without the sample.

### 2.9. Profiling of Organic Acids by LCMS

The CS of the strain was used for LCMS analysis. 500 μL of supernatant was filtered using a Whatman syringeless filter with a 0.45 mm pore size. A sample volume of 500 μL was diluted 20 times with mobile phase, filtered for 1 mL, and then 4 µL (sample), was injected into an LCMS/MS analyzer (Waters UPLC H class system fitted with a TQD MS/MS system) for analysis. The mobile phase solvents were Solvent A; 10 mM ammonium acetate: acetonitrile (50:50, pH 8) and Solvent B; acetonitrile with 0.05% formic acid. The first gradient for 30 s, was composed of an aqueous phase (A; 100%) and an organic phase (B; 0%). The gradient was changed at 5 min to A; 95% and B; 5% held for 30 s. The system was then brought back to its starting settings for 6 min and kept for 1 min to allow for equilibration before the following injection (flow rate: 0.1 mL/min). The eluted organic acids were monitored using a PDA detector, and the UPLC column effluent was directly injected (TQD-MS/MS system) without being split beforehand. This technique assists in identifying and measuring organic acids.

### 2.10. Pass Pharmacological Analysis

Prediction of the pharmacological activity was done using the PASS online server (http://www.way2drug.com/passonline/ (accessed on 7 January 2023). It evaluates the potential of the input compounds to elicit a specified pharmacological impact [49]. The collected data were computed and classified as “Pa” and “Pi,” where “Pa” represents probable pharmacological activity and “Pi” represents probable pharmacological inactivity. Compounds having Pa values greater than Pi (Pa > Pi) are thought to be viable for a particular pharmacological property.

### 2.11. Molecular Docking Simulation

For the molecular docking simulation of yeast α-glucosidase, a homology-built protein model was used from the previous work of the authors [50,51]. The template search was performed for the sequence of *Saccharomyces cerevisiae* α-glucosidase MAL-32 (UniProt ID: P38158), before model building to find the highest percentages of alignment among the Protein Data Bank (PDB) nearest to the homology sequences (72% structural identity and 84% sequence similarity to RCSB PDB protein model 3AXH) using SWISS-MODEL (https://swissmodel.expasy.org/ (accessed on 8 January 2023)) [52]. Given that the constructed model had already been validated by the authors in early research, the same model was used in this investigation. In case of α-amylase, the RCSB PDB database (https://www.rcsb.org/ (accessed on 8 January 2023)) was used to retrieve the porcine pancreatin α-amylase x-ray crystal structure of (PDB ID: 1DHK).

The preparation of protein molecules and ligands for the molecular docking simulation was done according to the previous work of the authors Patil et al. JBSD1 JBSD2, using Autodock tools 1.5.6 [51]. The prediction of the inhibitor binding site of α-amylase was done according to the literature available, whereas, for α-glucosidase, the same inhibitor binding site predicted in the previous study was used [53,54]. A three-dimensional grid box with 40 Å edges consisting of the inhibitor binding pocket was stationed at the coordinates x = −6.718 Å, y = −7.124 Å, and z = −18.281 Å for α-glucosidase. Similarly, for α-amylase the grid box with the same measurement was coordinated at x = 99.182 Å, y = 35.192 Å, and z = 18.991 Å. Subsequently, two-dimensional structures of RAMULAB54 organic acids were drawn and optimized for better orientation compatibility in a three-dimensional area using ACD ChemSketch. For both α-amylase and α-glucosidase, acarbose was used as the positive control [55]. The prepared protein and ligands compounds were docked using AutoDock Vina 1.1.2.

### 2.12. Molecular Dynamics Simulation

After the docking investigations, the lead compounds obtained were further studied to analyze their binding stability and conformational dynamics at the atomic level using molecular dynamics (MD) simulation. The MD simulation was performed for 100 ns according to Patil et al. using GROMACS-2018.1 [56]. Briefly, the pdb2gmx-assisted conversion of the protein-ligand complex and apo-protein molecules was followed by assigning a CHARMM36 force field to get the topological details. Similarly, the SwissParam server (https://www.swissparam.ch/ (accessed on 10 January 2023)) was used get the ligand topology details [57]. The optimal salt concentration (0.15 M) of the simulation box of the TIP3P water model edged 10 Å was maintained with the supplementation of Na^+^ and Cl^−^ counter ions, which resulted in the neutralization of the entire simulation system. This was followed by an energy minimization procedure of 50,000 steps of the steepest descent approach. The simulation systems were further equilibrated in two phases—namely NPT and NVT ensembles (1000 ps each), which were then proceeded with the MD simulation for 100 ns at a 310K-1 bar temperature-pressure configuration. The MD trajectories obtained at the end of the simulation including Root Mean Square Deviation (RMSD), Root Mean Square Fluctuation (RMSF), Radius of Gyration (Rg), and SASA (Solvent Accessible Surface Area), and hydrogen bonds were analyzed and plotted using QtGRACE software (Version 26.0).

### 2.13. Binding Free Energy Calculations

The determination of binding free energy responsible for protein-ligand complex formation was done using the Molecular Mechanics/Poisson-Boltzmann Surface Area (MM-PBSA) approach using the G MMPBSA tool, a plugin of GROMACS 18.1. The calculation of binding free energy was done using three components: molecular mechanical energy, polar solvation energy, and apolar solvation energy. For the analysis, MD simulation frames of the last 50 ns simulation run were used. The categories of binding free energy calculated were, Van der Waal’s energy (VDWE), electrostatic energy (EE), polar solvation energy (PSE), solvent-accessible surface area (SASAE) energy, and binding energy (BE). 

### 2.14. Statistical Analysis

The tests carried out in this study were performed in triplicates, and their outcomes were expressed as mean ± standard deviation. Duncan’s Multiple Range Test was employed after a one-way analysis of variance (ANOVA) to compare the isolates statistically using SPSS software (Version 21.0, Chicago, IL, USA). If the *p*-value was ≤0.05, the results were judged as statistically significant. Graph pad Prism version 8.0 was used to create the graphs (GraphPad Software Inc., San Diego, CA, USA).

## 3. Results and Discussion

### 3.1. Preliminary Biochemical Characterization

In this study, about 124 distinct morphic colonies, including 70 Gram-negative colonies and 54 Gram-positive colonies, were present in fermented sugarcane juice. About nine isolates with rod shape, catalase-negative, tolerance up to 4% NaCl, with optimal growth temperature (37 °C), and pH 7.4 were chosen for investigation (Table 1). Since the goal of this investigation was to identify the LAB, the ability to withstand harsh conditions was essential. As a result, the ability to withstand salt, temperature, and phenolic conditions was investigated. The RAMULAB54 could tolerate the temperature of 50 °C for 30 min where partial growth was observed. MYSRD108 and MYSRD71 strains isolated from fermented food vellappam had similar capabilities to tolerate these conditions [58]. Furthermore, phenol is a bacteriostatic substance produced by gut microorganisms that deaminate aromatic amino acids in the stomach [34]. All the isolates had remarkable tolerance to phenol (0.4%) over 24 h of incubation, and their cell counts varied from 7.23 to 8.47 CFU/mL. Also, the pH tolerance of the isolates showed a reduction in cell number as the pH became acidic (Table 2). Narendranath and Power (2005) discovered that the specific growth rate of *Lactobacilli* spp. in the manufacture of ethanol is noticeably low when the pH drops [59]. The isolate’s fermentation ability varied with RAMULAB54 showing fermentation of carbohydrates for eight of the sugars tested but negative for arabinose and starch, as shown in Table 3.

### 3.2. Molecular Identification of LAB

**Figure 1:**
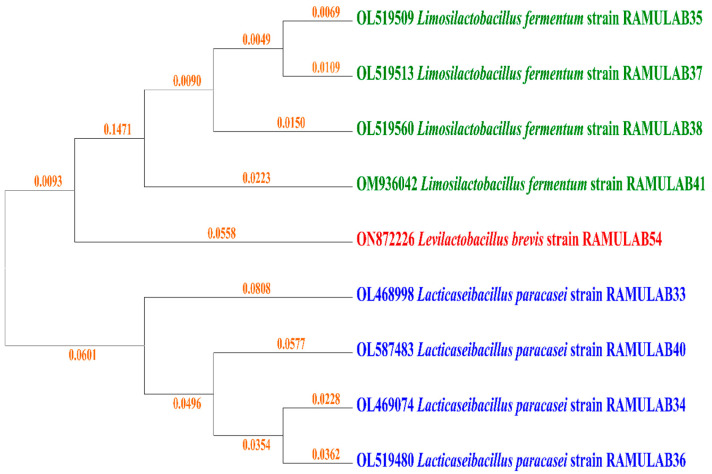
Phylogenetic tree comparison of strains isolated from fermented sugarcane juice based on maximum likelihood bootstrap analysis of 16S rRNA.

The fermented sugarcane isolates were amplified and the obtained sequence length varied from 1164–1564 bp. The homology for all the isolates had above >95% similarity and the NCBI GenBank accession numbers are shown in Figure 1. The NCBI blast analysis presented RAMULAB35, RAMULAB37, RAMULAB38, and RAMULAB 41 had similarity > 98% to *Limosilactobacillus fermentum*. The RAMULAB33, RAMULAB34, RAMULAB36, and RAMULAB40 expressed a similarity > 97% to *Lacticaseibacillus paracasei.* The RAMULAB54 had >99% similarity to *Levilactobacillus brevis*. *Limosilactobacillus* spp. and *Levilactobacillus* spp., according to examinations of the 16S rRNA gene sequences, were divided into separate groups (Figure 1).

### 3.3. Probiotic Properties

#### 3.3.1. Adherence Assay

##### Hydrophobicity, Autoaggregation, and Coaggregation of Fermented Sugarcane Juice Isolates

**Figure 2:**
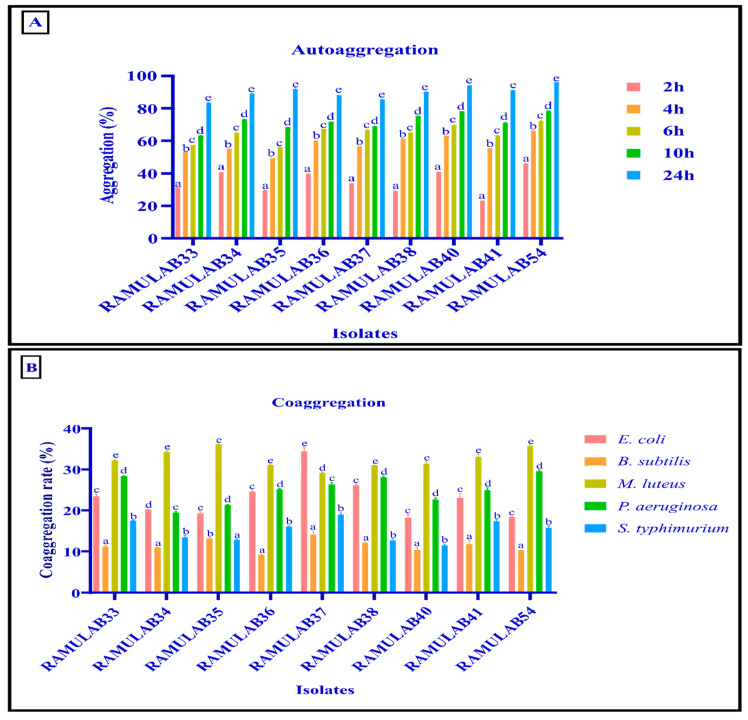
(**A**) The percentage of strains that autoaggregate over time at room temperature; (**B**) the percentage of LAB strains that coaggregate after two hours at room temperature. The mean ± SD is used to express data. According to the Duncan multiple range tests, the means in aggregation for 2 h with the superscripts (a–e) are significantly different (*p* ≤ 0.05).

LAB strains need to colonise the intestine’s surface in order to cling to pathogens and exhibit their abilities. Therefore, the ability of bacterial colonies from the same species to autoaggregate and be hydrophobic aids in the adhesion of microorganisms to the gut layer [60]. Bacterial interactions that are hydrophobic play important roles in adhesion and biofilm formation [61]. Xylene was used to measure the hydrophobicity of the cell surface. The fermented sugarcane juice isolate’s hydrophobicity ranged from 56.12–75.23% (Table 4). For bacterial colonization and protection, probiotic autoaggregation is necessary. Isolates in the current study expressed intensified autoaggregation as the incubation period was prolonged. Each isolate’s autoaggregation increased by approximately 55.07% from that exhibited in comparing the results shown after 24 h of incubation to those seen after 2 h (Figure 2A). The outermost surface of microorganisms has a hydrophobic nature that has been linked to the property of bacterial attachment to its host tissue; this characteristic may provide a competitive advantage that is crucial for bacterial maintenance in the human gastrointestinal tract [62]. According to several earlier reports, strain-dependent adhesiveness with hydrocarbons has also been found, which is consistent with our findings [37,38,60].

The ability of the isolates to coaggregate was tested and it showed that all the isolates had higher coaggregation capability with *M. luteus* and consecutively moderate with *E. coli* and *P. aeruginosa*. Comparatively, *B. subtilis* and *S. typhimurium* have lesser coaggregation ability as shown in Figure 2B. A similar gradual increase in the autoaggregation and maximum coaggregation with *E. coli* results was obtained for the *L. salivarius* M2-71 in the study carried out by Li et al. [63]. Coaggregation may be essential in the process of removing pathogens from the digestive system, according to Todorov et al. [64]. Through coaggregation, *Lactobacillus* strains can create a barrier that stops pathogenic bacteria from colonizing [37]. By coaggregating with a potential pathogen, the probiotic strain can release antimicrobial compounds that may stop the spread of pathogenic strains in the gastrointestinal system. Additionally, these characteristics may be used in the initial selection of probiotic microorganisms [65,66].

##### Adhesion to HT-29 Cell Lines and Buccal Epithelial Cells

**Figure 3:**
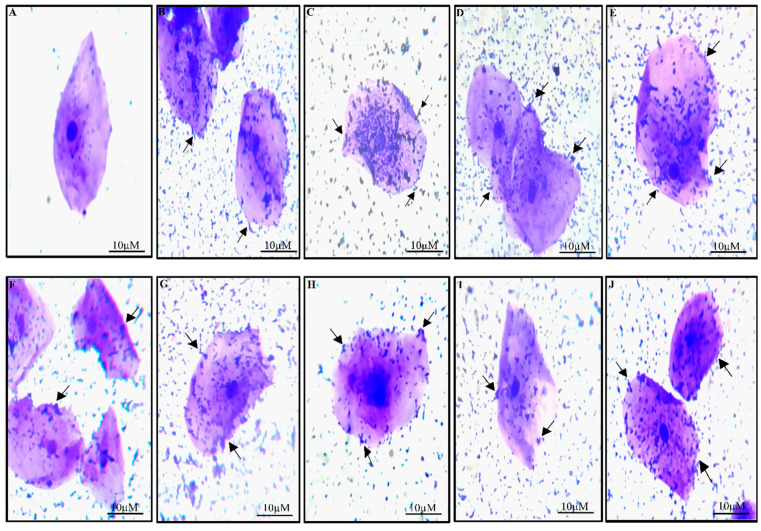
Under a light microscope, LAB strain adherence to buccal epithelial cells has been observed. The buccal epithelial cells in (**A**) are the control, and the isolates’ (**B**) RAMULAB33, (**C**) RAMULAB34, (**D**) RAMULAB35, (**E**) RAMULAB36, (**F**) RAMULAB37, (**G**) RAMULAB38, (**H**) RAMULAB40, (**I**) RAMULAB41 and (**J**) RAMULAB54 adhesion to these cells is shown. The arrow points to the isolates that are attached to the epithelial cells.

An advantageous characteristic of probiotic microorganisms is adhesion to the intestinal mucosa, which has been linked to many of their health advantages. Contact between the bacterial cell membrane and the other surfaces occurs during a complicated process known as cell adhesion [67]. Interest in developing in vitro models for the initial screening of potentially adhering strains has been sparked by challenges observed when examining bacterial adhesion in humans in particular [68]. The study using the buccal epithelial cells has helped us understand the adhesion capability of the LAB [34,69]. According to our research, the isolates’ capacity to adhere to buccal epithelial cells varied between 60 and 180 bacterial cells per epithelial cell. The capacity of all the isolates to attach to the epithelial cells was optimal (Figure 3). The adherence of LAB to epithelial cells was consistent with earlier studies [34,38,69].

The gut epithelium may be studied using the ideal model, the HT-29 cell line, a human colonic adenocarcinoma cell that after differentiating has structural characteristics of mature enterocytes [68]. As a result, isolate adhesions were seen with HT-29 cells; these adhesions were greater than the 54.15% represented in Table 5. The RAMULAB54 strain had the highest adhesion capability of 88.56% with HT-29 cells. Dhanani et al. in their investigation on *Lactobacillus plantarum* CS24.2 adhesion properties to HT-29 cells not only expressed adhesive properties but also prevented pathogen (*E. coli*) adhesion [67]. Similarly, the *L. paracasei* spp. investigated by Fonseca et al. expressed a 5% higher adhesion potential to HT-29 cells compared to the positive control used [70]. In the context of their successful colonisation, the capacity of LAB to stick to epithelial cells and mucosal surfaces has been recognised as a crucial characteristic. Due to the fact that our isolates exhibited increased adhesion with HT-29 cells, we may conclude that the cells can attach and express necessary activity.

#### 3.3.2. Tolerance Assay

##### Tolerance for Bile Salt in Acidic Conditions

**Figure 4:**
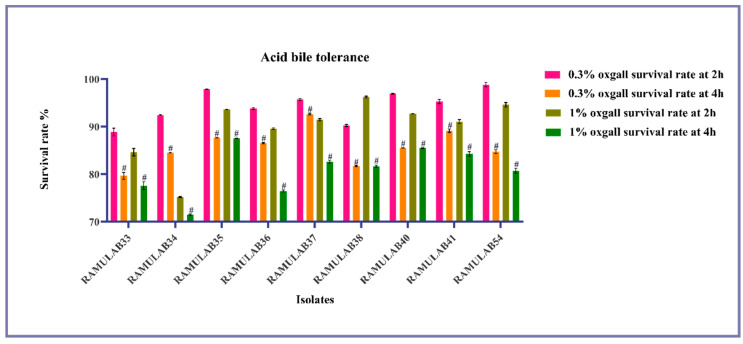
Shows the survival of LAB strains in acidic pH2 circumstances and various bile salt conditions, obtained from a fermented sugarcane juice sample with bile salt concentration parameters 0.3% and 1% for 2 and 4 h (37 °C) in MRS agar plates. The mean and SD are used to express data. Duncan multiple range tests reveal a significant difference (*p* ≤ 0.05) between the stated averages of the survival rate with a 2 h time interval and superscripts (#).

It is necessary that the isolates need to colonize with a longer transit time in order for their physiological activities to express optimally. The isolates must be able to endure digestion and withstand gastro conditions for up to 3 h and intestinal conditions for 3–8 h, with pH levels ranging from 2–8 [40]. In our study, the isolates effectively withstood an acidic environment (pH 2) and ox gall concentrations of 0.3 and 1%, according to the analysis of survival (Figure 4). For 4 h, the isolates can tolerate and show survival of 79.64–92.64% at 0.3% ox gall concentration and 71.45–87.56% at 1% ox gall concentration. At 1% ox gall concentration, RAMULAB34 showed the least reduction of 3% at 4 h. The higher ability of survival was observed in RAMULAB54 in both ox gall concentrations. As a result, it was determined that a decrease in survival rate was correlated with an increase in bile concentration.

##### Simulated Gastrointestinal Juice Tolerance Assay

**Figure 5:**
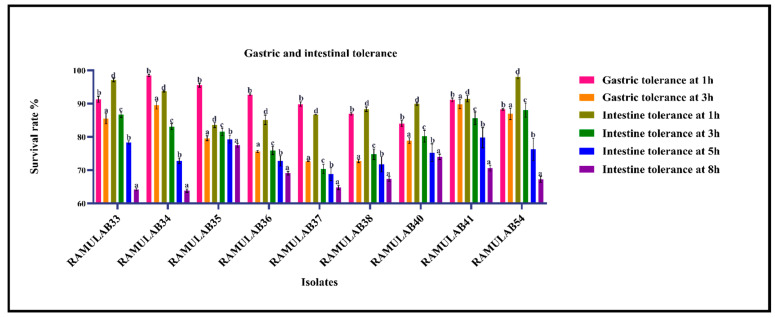
Shows the isolate survival rates in gastric and intestine juice. The mean ± SD are used to express data. According to the Duncan multiple range test, the means of the survival rates for the time intervals (1 h, 3 h, 5 h, and 8 h) are indicated with distinctive superscripts (a-e) are significantly different (*p* ≤ 0.05).

The ability of the isolates to grow at their best was expressed in this investigation by the gastrointestinal juice tolerance test. The survivability when subjected to gastric condition observed was above 72% for 3 h incubation. The intestinal tolerance for 8 h expressed survivability of above 64%. Figure 5 depicts the gastrointestinal survivability rate of the isolates. *L. casei* Zhang, in contrast, demonstrated a 63–69% survival rate in simulated gastric and intestinal juice conditions (pH 2.5), although a higher survival rate (>90%) was noted when delivered via fermented soymilk milk and bovine milk [71]. Vidhyasagar et al. observed that after 4 h of incubation, the putative probiotic *P. pentosaceus* VJ13’s cell number fluctuated and its survival rate dropped by 20% when exposed to gastric juice and intestinal juice [72]. If the isolates can withstand a harsh gastrointestinal environment, they are thought to be effective.

### 3.4. Safety Assessments

Another crucial element in preserving a balanced microbial ecosystem in the digestive system is the antibacterial activity of probiotic strains against pathogens. In this investigation, the isolates had their antibacterial efficacy against the pathogenic bacteria assessed. The isolates were able to inhibit every pathogen examined, except for *S. typhimurium* and *K. aerogenes*, as shown in Table 6, which ranged from 6 to 18 mm. Against *M. luteus* all the isolates displayed the highest levels of inhibition, whereas the isolates displayed lowest inhibition against *B. cereus* (Table 6). In line with this, Jiang et al. investigated *L. plantarum-derived* plantaricin NC8 had the ability to disrupt the cell membrane of *M. luteus*. Probiotics, therefore, function through a number of processes, one of which is the production of antimicrobial compounds [73]. On the other hand, it has been shown that *L. rhamnosus* can compromise the integrity of cellular membranes and result in ATP efflux, which causes hole formation and inhibits the development of *M. luteus* [74]. Typically, the probiotic benefits of *Lactobacilli* spp. are enhanced by the possible generation of antimicrobial substances such as lactic acid, superoxide radicals, and/or antimicrobial peptides such as bacteriocins [75,76,77].

### 3.5. Antibiotic Sensitivity

The isolates are probiotics that are widely regarded with the status of generally recognized as safe (GRAS). However, it is advised that putative probiotics be examined for safety using primary testing, which includes antibiotic resistance patterns. All the fermented sugarcane juice isolates were subjected to determine antibiotic susceptibility or resistance. Among the six antibiotics tested, the isolates were resistant to vancomycin and methicillin and susceptible to streptomycin, tetracycline, azithromycin, and, ampicillin. The acquired findings’ chart was contrasted with the reference standard chart (Table 7). The majority of experts thought that acquired and endogenous resistance were both a result of protracted evolutionary processes that led to the development of resistance [43]. The findings of the tests for antibiotic susceptibility show that lactic acid bacteria are inherently resistant to vancomycin, which is consistent with the majority of earlier studies done on various traditional fermented foods [78,79].

### 3.6. Hemolytic Assay

Lack of hemolytic activity is beneficial when preferring probiotic strains for safety reasons since such strains are non-virulent and the lack of hemolysin ensures that virulence will not emerge among the bacterial strains (FAO/WHO 2006). It has been proposed that a fundamental component of pathogen virulence is the development of the enzymes necessary to break down mucin. Therefore, this trait is not recommended for probiotic strains since it changes the intestinal mucosal lining, which makes it easier for infections and other toxic substances to infiltrate the mucosa [81]. The absence of hemolytic action is essential for the safety of probiotics. The γ-hemolysis representing no zone around the colonies shows that the organism is safe. All nine isolates had γ-hemolysis activity that was demonstrated as a clear zone indicating that the organism did not show hemolysis. *S. mutans* which was considered as negative control in this study expressed the β-hemolysis that is the complete breakdown of red blood cells around the colonies. Our findings concurred with those of Oh and Jung, who identified six *Lactobacillus* spp. isolated from fermented millet-based alcoholic beverages that showed γ-hemolysis [82]. Results by Wang et al. who assessed the probiotic potential of lactic acid bacteria from infant feces found no evidence of probiotic’s hemolytic activity and likewise compatible with the findings of the current study [83]. The fermented sugarcane juice strains lack hemolysin and are not pathogenic.

### 3.7. Antioxidant Assay

**Figure 6:**
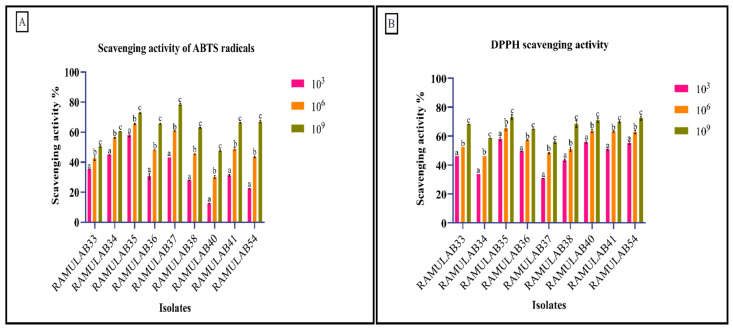
Shows of the isolates’ (**A**) capacity for scavenging ABTS radicals and (**B**) capacity for scavenging DPPH free radicals. The mean and SD are used to express data. According to the Duncan multiple range test, the means of the scavenging activity of various CFU/mL with distinct superscripts (a–c) are significantly different (*p* ≤ 0.05).

In addition to traits examined for probiotic qualities, functional traits of *Lactobacillus* spp., like antioxidant capacity, are important to assess. Oxidative stress is caused by an imbalance between the generation of reactive oxygen species and antioxidant defenses [45]. The most harmful reactive oxygen species are hydroxyl and related radicals, which cause oxidative damage to biomolecules. From DPPH and ABTS, antioxidants’ electrons or hydrogen atoms are removed and transformed into compounds that are constantly stable [84]. According to some research, probiotic bacteria’s capacity to create antioxidants may help reduce free radicals, which would lower oxidative stress [42,85]. In our investigation, the scavenging activity of ABTS and DPPH of the isolates gradually increased as the cell count increased (CFU/mL). At 10^9^ CFU/mL, the range of ABTS scavenging activity was found to be 47.73–78.40%. For DPPH scavenging activity at 10^9^ CFU/mL, cells expressed a 55.73–73.06% range of activity (Figure 6). The results that we obtained are comparable to *Lactobacillus* spp. which was studied for its antioxidant capability by Kim et al. against the ABTS and DPPH for 10^8^ CFU/Ml [86]. According to Chooruk et al., individual activities differed within/between strains and species, showing that the strains differed in their antioxidative capabilities [87].

### 3.8. Inhibitory Assay for the Carbohydrate Hydrolyzing Enzymes (α-Glucosidase and α-Amylase)

**Figure 7:**
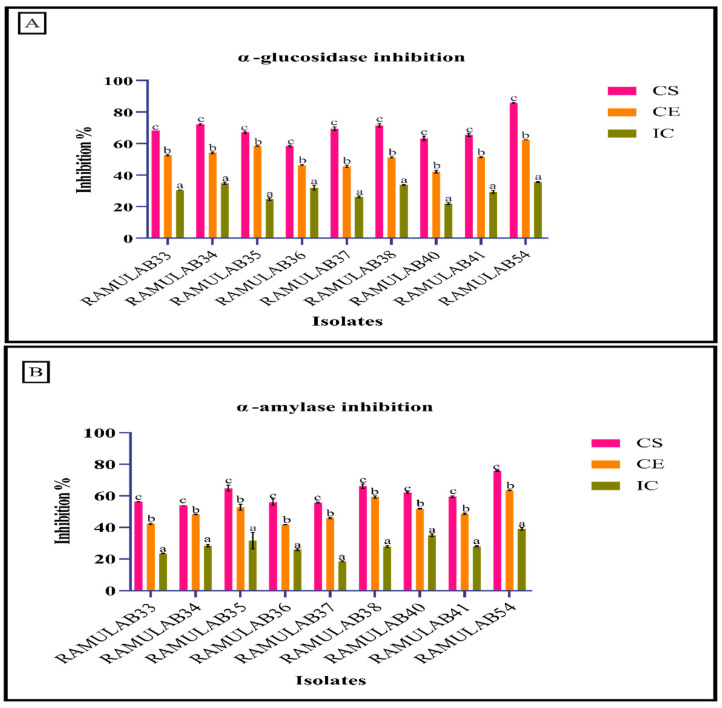
Shows the isolates’ ability to hinder the enzymes α-glucosidase (**A**) and α-amylase (**B**). The mean and SD are used to express data. The means of the inhibitory activity of the CS, CE, and IC with the various superscripts (a–c) are substantially different (*p* ≤ 0.05), according to the Duncan multiple range test.

This study’s main goal was to assess the efficacy of probiotic bacteria isolated from fermented sugarcane juice in inhibiting the enzymes responsible for hydrolyzing carbohydrates (α-glucosidase and α-amylase). Although α-glucosidase and α-amylase inhibitors are often utilized in clinical research, novel inhibitors are always being studied in an effort to minimize side effects and medication costs. Numerous natural, plant-based, and food-based sources have been researched, as well as functional foods with inhibitory effects [88,89,90,91]. These two enzymes are responsible for transforming oligosaccharides and disaccharides into assimilable monosaccharides. A slower rate of glucose absorption and a lower postprandial plasma glucose level are the results of these enzyme inhibitions, which slow down the digestive process and extend the time required to break down included carbohydrates [1,6]. Numerous sources of *Lactobacillus* spp. have been examined to study more about this suppression of the enzymes that break down carbohydrates [92,93]. Our investigation used the isolates’ derivatives CS, CE, and IC for the isolates to perform inhibitory activity against α-glucosidase and α -amylase. The isolates had a higher inhibitory potential when tested using CS over that of CE and IC, ranging between 21–85% against yeast α-glucosidase and 18–75% for α-amylase (Figure 7). The CS of *Levilactobacillus brevis* RAMULAB54 among other isolates had the maximum inhibition; it inhibited yeast α-glucosidase by 85.88% and α-amylase by 75.89%. This is in line with studies from Chen et al. *L. casei* 2W and *L. rhamnosus* Z7, and Son et al., who found that *L. brevis* KU15006 expressed possible probiotic characteristics and had higher α-glucosidase inhibitory activity in supernatant (cell-free) than in intact cells or the extract [94,95]. Also, Huang et al., in their investigation of the exopolysaccharide made from *L. plantarum* H31, found that its α-amylase inhibitory activity varied for crude and pure fractions [96]. Son et al. expressed that *L. brevis* KU15006 has shown stronger α-glucosidase inhibitory efficacy than the commercially available LAB [95]. *L. casei* 2607, *L. acidophilus* 33,200, *L. delbrueckii* ssp. *bulgaricus1092* were found by Ramchandra et al. to express higher than 80% inhibitory activity against α-glucosidase [97]. The investigation by Chen et al. found that the cell-free extract was engrossed from the small intestine into the blood whereas the intact cells were unable to do so [94]. This shows that inhibitory substances are present in the extract and supernatant from cell-free samples, whereas the intact cell contains the least inhibitory substances.

### 3.9. Profiling of Organic Acid

A complex combination of metabolic products, including enzymes, peptides, fatty acids, amino acids, vitamins, secreted proteins, organic acids, etc., can be produced by probiotics in the cell-free supernatants. The LAB has the ability to convert organic acids derived from carbohydrate substrates into a wide range of metabolites. Lactate, succinate, formate, acetate, and citrate are known organic acids produced by LAB [98]. As per this study’s results and on a comparative note, the strain RAMULAB54 showed a higher inhibitory ability for both α-glucosidase (85.88%) and α-amylase (75.89%). Thus, the organic acid determination was carried out for RAMULAB54 which expressed the organic acid present as shown in Table 8. Hydroxycitric acid, citric acid, and lactic acid were detected to be present in the highest concentrations over the other acids. Basa et al., in their investigation, show that evaluation of *L. plantarum* for their organic acid level revealed that it was high in oleic acid content [99]. According to Zalan et al., the species and the media have an impact on how much organic acid is produced. Traditionally, organic acids from LAB help to stop food from spoiling and improve taste [100]. As natural preservatives and potential antibiotic substitutes, organic acids play an important role in food and feed. They have the potential to be both bacteriostatic and/or bactericidal, and generally, as pH drops or when other antimicrobial substances produced by the bacteria are present, their efficacy rises. The present study is mainly focused on determining the capability of the organic acids with potential inhibitory activity against the carbohydrate hydrolyzing enzyme.

### 3.10. Pass Pharmacological Potential Analysis

The PASS analysis findings show that each of the compounds has significant anti-diabetic activity (Table 9). Especially, hydroxycitric acid and tartaric acid showed high Pa, which indicates that their probable pharmacological activity is greater than other compounds.

### 3.11. Molecular Docking Studies

Molecular docking was completed using AutoDock Vina and the mechanism of interaction between organic acid compounds (RAMULAB54 derivatives) and the target proteins (α-glucosidase and α-amylase) were studied using BIOVIA Discovery Studios Visualizer 2021. The results of this virtual screening, which was based on parameters such as the total number of intermolecular interactions, the total number of hydrogen bonds, and the binding affinity are detailed in Table 10. The highest docking score of −5.8 kcal/mol and −5.4 kcal/mol were shown by hydroxycitric acid against α-glucosidase and α-amylase, respectively. Comparatively, the acarbose control showed the lowest docked score of −5.6 kcal/mol and −5.4 kcal/mol against the target proteins.

**Figure 8:**
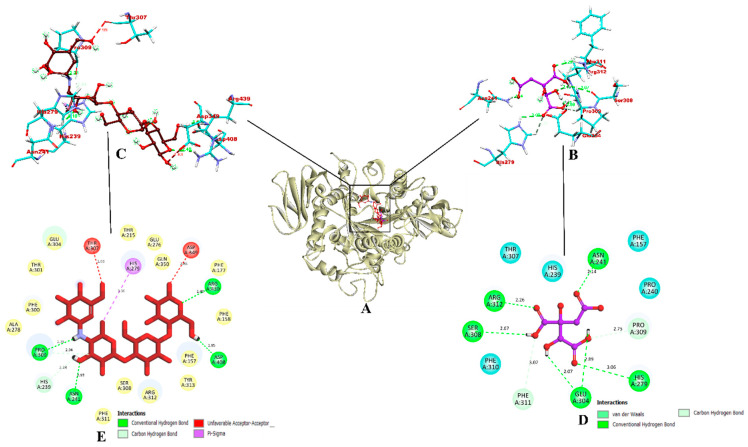
The binding interactions of hydroxyacetic acid and acarbose within the inhibitor binding site of target protein α-glucosidase. (**A**) Ribbon representation of a protein model bound with the ligands (inside the binding site); (**B**,**C**) Three-dimensional binding pattern of hydroxyacetic acid and acarbose, respectively. (**D**,**E**) Two-dimensional binding pattern of hydroxyacetic acid and acarbose, respectively. Red: acarbose; purple: hydroxyacetic acid. Blue and yellow: surrounding residues; colored: bound residues.

Concordant to Maradesha et al. [50], the inhibitor binding site is located in domain A, consisting of the catalytic residues required to bind with the ligand. The docked structure was visualized to show the hydroxycitric acid interacts with important amino acid residues of α-glucosidase, such as ASN241, ARG312, GLU304, SER308, HIS279, PRO309, and PHE311 via hydrogen bonding. Comparatively, acarbose formed only seven non-bounded interactions out of which six were of hydrogen bond and one hydrophobic bond of pi-sigma. The hydrogen bonds were formed via ASN241, ARG439, ASP408, PRO309, HIS239, and HIS279 forming one hydrophobic bond of pi-sigma and the unfavorable acceptor-acceptor bonds were formed via THR307 and ASP349; see Figure 8. The results obtained in this study are in accordance with the previous studies by Patil et al. [49] and Prabhakaran et al. [98], with respect to the binding interactions of the ligands with the residues from the inhibitor binding site. Since hydroxycitric acid binds to the same inhibitor binding region reported in these studies and ligands reported in these studies show in vitro effect, hydroxycitric acid from our study is expected to be a potential inhibitor of α-glucosidase protein.

**Figure 9:**
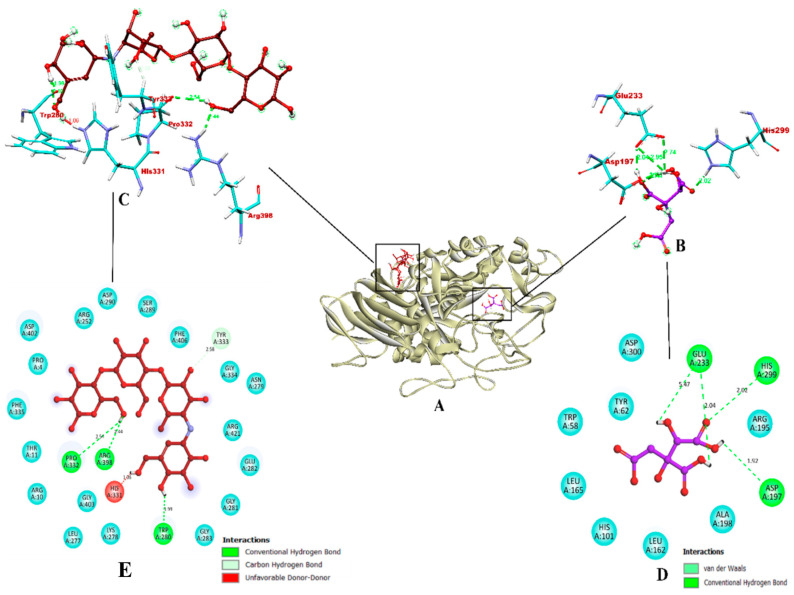
The binding interactions of hydroxyacetic acid and acarbose within the inhibitor binding site of target protein α-amylase. (**A**) Ribbon representation of a protein model bound with the ligands (inside the binding site); (**B**,**C**) Three-dimensional binding pattern of hydroxyacetic acid and acarbose, respectively. (**D**,**E**) Two-dimensional binding pattern of hydroxyacetic acid and acarbose, respectively. Red: acarbose; purple: hydroxyacetic acid. Blue: surrounding residues; colored: bound residues.

In the case of α-amylase, the ligand was found to be bound within the binding region occupied by the co-crystal ligand. The interaction shows that the hydroxycitric acid is bound to the key residues of α-amylase (GLU233 and ASP197) [101,102]. According to Patil et al. [102] and Shivanna et al. [103] study, catalytic residues Asp197 and Glu233 bound with hydrogen bond form a strong inhibitory activity and might substantially reduce α-amylase activity. However, acarbose-α-amylase formed a total four hydrogen bonds and ligand and one unfavorable bond was formed between HIS331 and ligand. Figure 9 depicts the binding interaction between hydroxycitric acid and acarbose with α-amylase. The interaction results indicated that hydroxycitric acid is comparatively better than acarbose and may act as the better inhibitor.

### 3.12. Molecular Dynamics Simulation

Although the molecular docking approach was used to understand the interactions between hydroxycitric acid and acarbose with α-glucosidase and α-amylase proteins, the differences in interaction between the compound and the proteins does not depict the stability and structural flexibility of the protein-bound ligand complexes. Thus, to validate the docking result of hydroxycitric acid with α-glucosidase and α-amylase, molecular dynamics (MD) simulation was conducted.

**Figure 10:**
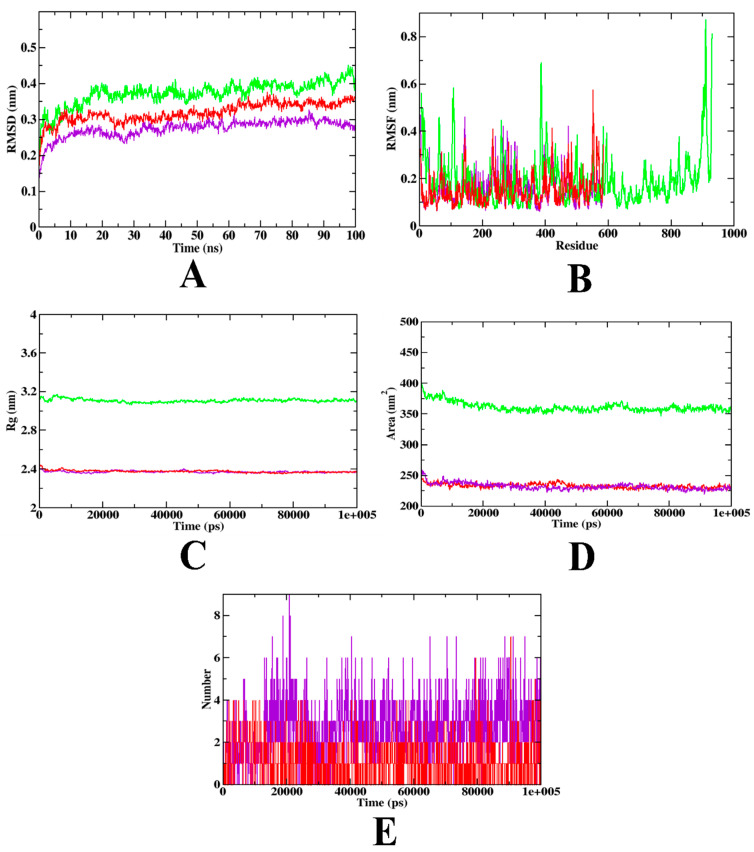
MD simulation trajectories plotted to show the comparative stability of ligands inside the inhibitor binding site of α-glucosidase (**A**) RMSD, (**B**) RMSF, (**C**) Rg, and (**D**) SASA, and (**E**) Ligand H-Bonds. Red: protein-acarbose; purple: protein-hydroxyacetic acid complex, and green: apo-protein.

The MD trajectory values for the simulation conducted for α-glucosidase complexed with acarbose and hydroxycitric acid have been depicted in Table 11, and the same trajectories have been combined and visualized in the form of graphs in Figure 10. Based on the analysis, both the complexes and apoprotein reached equilibrium after 25 ns, and it can be said that hydroxycitric acid stayed inside the inhibitor binding site throughout the simulation period. Compared with the acarbose, the hydroxycitric acid complex reached equilibrium more rapidly, thus, the hydroxycitric acid complex showed the best stability. On the other hand, RMSF analysis was conducted to examine the binding efficiency of hydroxycitric acid complex with α-glucosidase along with control acarbose, values for all the residues were measured based on 100 ns trajectory. The RMSF fluctuation of both acarbose and hydroxycitric acid-bound complexes ranges between 0.1 Å–5 Å. The plot evaluation indicates the target protein’s minimal fluctuation and comparable secondary conformational stability when bound to compounds. Acarbose-bound complex was found to have more fluctuations, indicating its comparative instability. To evaluate the change of protein structure during complex formation the Rg plot was analyzed. The Rg values of hydroxycitric acid and acarbose complexes kept fluctuating at 2.4 nm, indicating compactness their binding. The SASA plot was evaluated to predict the conformational change in the binding region [104]. The SASA of hydroxycitric acid and acarbose fluctuated within the same range of 240~250 nm^2^, respectively. Finally, the ligand hydrogen bond was analyzed to understand the structural reconstruction. Based on the plot it can be seen that the complex may have undergone structural modification and when compared with acarbose, hydroxycitric acid formed more H-bonds with protein during the 100 ns simulation, indicating that the hydroxycitric acid complex was more stable than the other. The results were in accordance with the previous results of the MD study conducted by Ganavi et al. [105].

**Figure 11:**
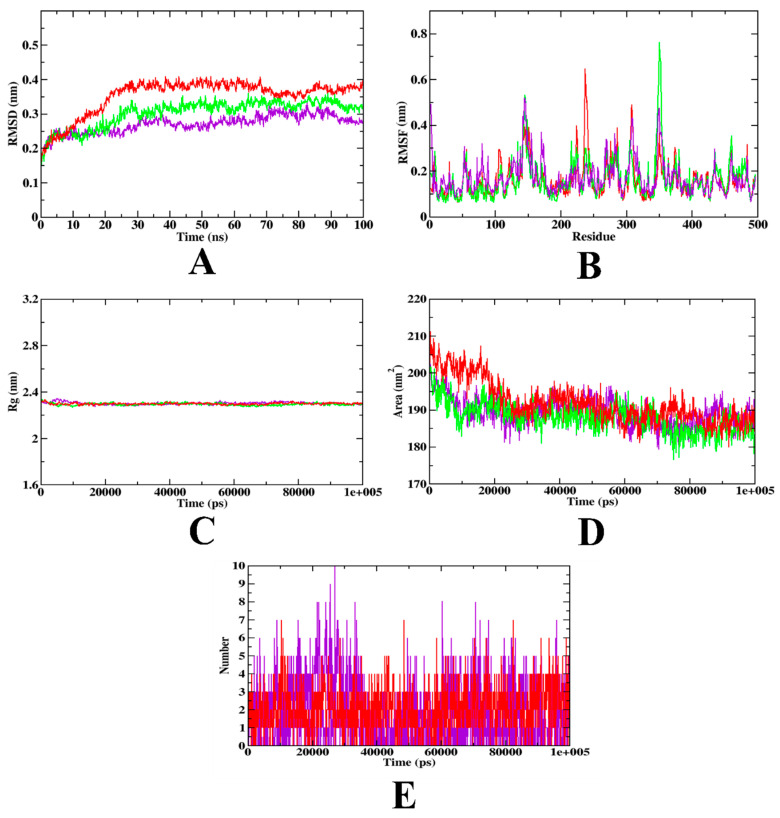
MD simulation trajectories plotted to show the comparative stability of ligands inside the inhibitor binding site of α-amylase (**A**) RMSD, (**B**) RMSF, (**C**) Rg, and (**D**) SASA, and (**E**) Ligand H-Bonds. Red: protein-acarbose; purple: protein-hydroxyacetic acid complex, and green: apo-protein.

In the case of α-amylase, the RMSD plots depict that the hydroxycitric acid complex and the apo-protein were found to be within the range of 0.20–0.30 nm. The acarbose complex was found with a 0.25–0.35 nm range. Compared to the acarbose complex, hydroxycitric acid was found to be stable with minimal fluctuation throughout the simulation. In the RMSF analysis, both the hydroxycitric acid complex, acarbose complex, and the apo-protein atoms were on par, with more or less the same fluctuating behavior. Furthermore, Rg and SASA graphs were examined to demonstrate the structural compactness of the generated structure. The Rg analysis shows that the protein along with hydroxycitric acid and acarbose complex was found to be within the same range of 2.31 nm; similarly, the SASA value was determined to be comparable, with a similar pattern. The SASA of hydroxycitric acid and acarbose fluctuated within the same range of 190~200 nm^2^, respectively. Furthermore, the H-bond was examined to identify structural re-agreement, and it was evident that the complex has undergone conformational changes. However, in terms of ligand hydrogen bonding interactions, acarbose formed fewer hydrogen bonds than hydroxycitric acid. These results were in accordance with the previous study which used the same protein model (PDB ID: IDHK). Figure 11 depicts the illustration of the simulation and Table 12 depicts trajectory values for α -amylase.

### 3.13. Binding Free Energy Calculations

The most used approach for calculating free binding energies has been determined as MM/PBSA. The binding free energy study demonstrates that van der Waal’s energy and binding energy had an important impact in the development of protein-ligand complexes during MD simulation [106,107]. All hydroxycitric acid-free energy estimates were energetically viable. Comparably, acarbose-bound complexes had lower binding energy than hydroxycitric acid complexes, suggesting weakened interactions and binding affinity. Furthermore, the findings of binding affinity corroborate the theory of docking and dynamics simulations. Furthermore, the results appeared consistent with previous research that calculated binding free energy for α-glucosidase and α-amylase [51,108]. The values of binding free energy estimations generated using the MMPBSA approach are summarized in Table 13.

## 4. Conclusions

Previously, studies have shown the occurrence of *Lactobacillus* spp. in sugarcane fermentation and processing along with its usage for fermentation. Yet, this is the preliminary approach to isolate *Lactobacillus* spp. from fermented sugarcane juice with probiotic traits and exhibiting antidiabetic activity. The results of this study demonstrated that the LABs that were isolated were secure and had critical properties such as tolerance to bile salt and acid, gastrointestinal environment, remarkable adherence (autoaggregation, coaggregation capacities, and hydrophobicity abilities), antibiotic, antibacterial, and antioxidant activities. In this study, the strains isolated from fermented sugarcane juice effectively inhibited α-glucosidase and α-amylase. The study has therefore contributed to our comprehension of the LAB’s potential to inhibit the carbohydrate hydrolyzing enzyme and effectively treat post-prandial hyperglycemia. Consuming probiotic LAB can thereby improve gut health and reduce the risk associated with diabetes. Probiotics release a complex array of metabolic products, including organic acids, short-chain fatty acids, enzymes, proteins, amino acids, peptides, vitamins, and biosurfactants. However, LAB strains such as *Lactobacillus* spp. may offer advantages over pharmacological therapy due to their potential use as dietary supplements, medical foods, or biotherapeutics for diabetes. They may also have a wider spectrum of activity. *Lactobacillus* strains identified in this investigation should be evaluated using animal models to better understand the source of the bioactivity and their respective bioavailability.

## Figures and Tables

**Table 1:** The phenotypic characteristics and tolerability of the LAB strains isolated from the sample of fermented sugarcane juice.

	Tests	Gram Staining	Catalase	Morphology	Temperature (°C) *					NaCl Concentration (%) *				
					4	10	37	45	50	2	4	6	8	10
Isolates	RAMULAB33	Positive	Negative	Bacilli	A	A	P	A	A	P	P	A	A	A
	RAMULAB34	Positive	Negative	Bacilli	A	A	P	A	A	P	P	A	A	A
	RAMULAB35	Positive	Negative	Bacilli	A	A	P	A	A	P	P	A	A	A
	RAMULAB36	Positive	Negative	Bacilli	A	A	P	A	A	P	P	A	A	A
	RAMULAB37	Positive	Negative	Bacilli	A	A	P	A	A	P	P	A	A	A
	RAMULAB38	Positive	Negative	Bacilli	A	A	P	A	A	P	P	A	A	A
	RAMULAB40	Positive	Negative	Bacilli	A	A	P	A	A	P	P	A	A	A
	RAMULAB41	Positive	Negative	Bacilli	A	A	P	A	A	P	P	A	A	A
	RAMULAB54	Positive	Negative	Bacilli	A	A	P	*p*	*p*	P	P	A	A	A

* ‘A’ indicates the absence of growth ‘P’ indicates the presence of growth and ‘*p*’ indicates partial growth.

**Table 2:** Fermented sugarcane juice isolates phenol tolerance and growth at different pH expressed in CFU/mL.

	Phenol Tolerance (CFU/ mL) *		Growth at Different pH (CFU/ mL) *			
Isolates	0 h	24 h	2	4	6	7.4
RAMULAB33	9.81 ± 0.02 ^c^	8.08 ± 0.11 ^c^	6.24 ± 0.11 ^d^	7.13 ± 0.01 ^c^	9.73 ± 0.02 ^d^	9.89 ± 0.12 ^c^
RAMULAB34	8.66 ± 0.01 ^a^	8.15 ± 0.14 ^c^	6.02 ± 0.03 ^a^	7.16 ± 0.01 ^c^	8.96 ± 0.11 ^b^	9.16 ± 0.01 ^b^
RAMULAB35	9.78 ± 0.15 ^c^	7.25 ± 0.31 ^a^	6.12 ± 0.02 ^b^	7.32 ± 0.01 ^d^	8.78 ± 0.04 ^b^	9.98 ± 0.03 ^c^
RAMULAB36	9.84 ± 0.24 ^c^	8.25 ± 0.01 ^c^	6.19 ± 0.09 ^c^	6.41 ± 0.12 ^b^	8.21 ± 0.31 ^a^	9.71 ± 0.01 ^c^
RAMULAB37	9.12 ± 0.02 ^b^	8.44 ± 0.07 ^c^	6.00 ± 0.13 ^a^	6.14 ± 0.02 ^a^	8.99 ± 0.12 ^b^	9.39 ± 0.04 ^b^
RAMULAB38	9.65 ± 0.05 ^c^	7.89 ± 0.01 ^b^	6.13 ± 0.01 ^b^	7.05 ± 0.03 ^c^	8.85 ± 0.15 ^b^	9.95 ± 0.01 ^c^
RAMULAB40	9.11 ± 0.18 ^b^	7.29 ± 0.10 ^a^	6.15 ± 0.16 ^c^	7.25 ± 0.01 ^d^	9.15 ± 0.23 ^c^	9.45 ± 0.03 ^b^
RAMULAB41	8.81 ± 0.10 ^a^	7.23 ± 0.09 ^a^	6.11 ± 0.05 ^b^	6.91 ± 0.03 ^b^	8.81 ± 0.11 ^b^	9.01 ± 0.01 ^a^
RAMULAB54	9.91 ± 0.01 ^d^	8.47 ± 0.03 ^c^	6.23 ± 0.01 ^d^	7.99 ± 0.11 ^e^	9.89 ± 0.01 ^d^	10.11 ± 0.71 ^d^

* Data are presented as mean ± SD. According to Duncan’s multiple range test, means in the same column are denoted by various letters (a–e) and are significantly different (*p* ≤ 0.05).

**Table 3:** Fermented sugarcane juice isolates carbohydrate fermentation assay.

	Carbohydrates Fermentation									
Isolates	G	DX	LX	S	M	Mal	L	Gal	Ara	ST
RAMULAB33	+	-	+	+	-	+	-	-	-	-
RAMULAB34	+	-	+	+	+	+	+	+	-	-
RAMULAB35	+	+	-	+	-	+	-	-	-	-
RAMULAB36	+	-	+	+	+	+	+	+	-	-
RAMULAB37	+	+	-	+	+	+	+	+	-	-
RAMULAB38	+	+	-	+	+	+	+	+	-	-
RAMULAB40	+	+	-	+	+	+	+	+	-	-
RAMULAB41	+	+	-	+	+	+	+	+	-	-
RAMULAB54	+	+	+	+	+	+	+	+	-	-

‘-’ indicates the absence of growth, ‘+’ indicates the presence of growth; G: glucose, DX: D-xylose, LX: L-xylose, S: sucrose, M: mannitol, Mal: maltose, L: lactose, Gal: galactose, Ara: arabinose and ST: starch.

**Table 4:** Cell surface hydrophobicity of fermented sugarcane juice isolates.

	Cell Surface Hydrophobicity (%) *
RAMULAB33	56.12 ± 0.09 ^a^
RAMULAB34	70.54 ±0.05 ^d^
RAMULAB35	57.81 ± 0.01 ^a^
RAMULAB36	68.15 ± 0.09 ^c^
RAMULAB37	65.33 ± 0.12 ^b^
RAMULAB38	73.53 ± 0.04 ^d^
RAMULAB40	73.82 ± 0.05 ^d^
RAMULAB41	63.95 ± 0.04 ^b^
RAMULAB54	75.23 ± 0.06 ^d^

* The data are displayed as mean ± SD. The means in the same column represented by different letters (a–d) are substantially different (*p* ≤ 0.05), according to Duncan’s multiple range test.

**Table 5:** HT-29 cells adhesion assay of isolates from fermented sugarcane juice.

Isolates	HT-29 Adhesion (%) *
RAMULAB33	64.34 ± 0.11 ^b^
RAMULAB34	69.16 ± 0.19 ^c^
RAMULAB35	75.23 ± 0.08 ^d^
RAMULAB36	85.12 ± 0.05 ^e^
RAMULAB37	54.15 ± 0.01 ^a^
RAMULAB38	65.12 ± 0.03 ^b^
RAMULAB40	72.14 ± 0.06 ^d^
RAMULAB41	81.15 ± 0.04 ^e^
RAMULAB54	88.56 ± 0.03 ^f^

* The result values are expressed as Mean ± SD. Duncan’s multiple range test indicates that the means in the same column denoted by different letters (a–f) are significantly distinct (*p* ≤ 0.05).

**Table 6:** Antibacterial activity of the fermented sugarcane isolates.

Isolates		RAMULAB33	RAMULAB34	RAMULAB35	RAMULAB36	RAMULAB37	RAMULAB38	RAMULAB40	RAMULAB41	RAMULAB54
Pathogens	*K. pneumoniae*	*+*	*+*	*+*	*+*	*+*	*+*	*+*	*+*	*+*
	*E. coli*	*++*	*++*	*++*	*++*	*+++*	*++*	*++*	*++*	*+++*
	*S. typhimurium*	*-*	*-*	*-*	*-*	*-*	*-*	*-*	*-*	*-*
	*S. aureus*	*++*	*++*	*++*	*++*	*++*	*++*	*++*	*++*	*+++*
	*P. aeruginosa*	*++*	*+++*	*++*	*+++*	*+++*	*+++*	*+++*	*+++*	*+++*
	*B. cereus*	*+*	*+*	*+*	*+*	*+*	*+*	*+*	*+*	*+*
	*M. luteus*	*+++*	*+++*	*+++*	*+++*	*+++*	*+++*	*+++*	*+++*	*+++*
	*B. subtilis*	*+*	*+*	*+*	*+*	*+*	*+*	*+*	*+*	*+*
	*P. florescens*	*++*	*++*	*++*	*++*	*++*	*++*	*++*	*++*	*++*
	*K. aerogenes*	*-*	*-*	*-*	*-*	*-*	*-*	*-*	*-*	*-*

Zones of inhibition are indicated (mm): (-): no inhibition; (+): minimal (5); (++): adequate (>6); (+++): robust (>15).

**Table 7:** Based on the Clinical and Laboratory Standards Institute (CLSI, 2018)’s antibiotic susceptibility test results, isolates representing both resistance and sensitivity were examined [80]. Streptomycin (STR), tetracycline (TET), azithromycin (AZM), ampicillin (AMP) and methicillin (MET). The inhibitory zone (mm) of the appropriate antibiotics is where the sensitivity/resistance breakpoints are expressed.

Sl. No.	1	2	3	4	5	6
Antibiotic	Streptomycin (STR)	Vancomycin (V)	Tetracycline (TET)	Azithromycin (AZM)	Ampicillin (AMP)	Methicillin (MET)
The Inhibitory Zone (S/R mm)	(≥15/≤12)	(≥17/≤14)	(≥19/≤14)	(≥13/≤12)	(≥17/≤14)	(≥22/≤17)
RAMULAB33	S	R	S	S	S	R
RAMULAB34	S	R	S	S	S	R
RAMULAB35	S	R	S	S	S	R
RAMULAB36	S	R	S	S	S	R
RAMULAB37	S	R	S	S	S	R
RAMULAB38	S	R	S	S	S	R
RAMULAB40	S	R	S	S	S	R
RAMULAB41	S	R	S	S	S	R
RAMULAB54	S	R	S	S	S	R

**Table 8:** Organic acid profile of the RAMULAB54.

Organic Acids	RAMULAB54 (mg/mL)
Lactic acid	7.09 ± 0.18
Pyruvic acid	0.59 ± 0.09
Malonic acid	3.62 ± 0.14
Maleic acid	0.04 ± 0.00
Fumaric acid	0.05 ± 0.00
Succinic acid	6.68 ± 0.48
Malic acid	5.68 ± 0.11
Tartaric acid	0.03 ± 0.00
Shikimic acid	0.33 ± 0.03
Citric acid	12.27 ± 0.39
Hydroxycitric acid	14.05 ± 0.58

**Table 9:** Prediction of PASS result of RAMULAB54 derivatives.

Compound	Activity	Pa	Pi
Citric acid	Antidiabetic	0.648	0.009
Fumaric acid	Antidiabetic	0.512	0.021
Hydroxycitric acid	Antidiabetic	0.708	0.006
Lactic acid	Antidiabetic	0.680	0.007
Malic acid	Antidiabetic	0.639	0.009
Malonic acid	Antidiabetic	0.270	0.100
Pyruvic acid	Antidiabetic symptomatic	0.228	0.095
Shikimic acid	Antidiabetic	0.203	0.160
Succinic acid	Antidiabetic	0.440	0.034
Tartaric acid	Antidiabetic	0.719	0.005
Acarbose	Antidiabetic	0.693	0.007

**Table 10:** Virtual screening of RAMULAB54 derivatives against α-glucosidase and α-amylase (PDB ID: 1DHK).

Compound	BA (kcal/mol)		TIN		THB	
	AG	AM	AG	AM	AG	AM
Citric acid	−5.4	−5.4	6	5	6	5
Fumaric acid	−5.3	−4.2	6	2	6	2
Hydroxycitric acid	−5.8	−5.5	8	4	8	4
Lactic acid	−4.5	−3.6	3	4	3	3
Maleic acid	−5.3	−4.1	5	4	5	4
Malic acid	−5.2	−4.4	4	3	4	3
Malonic acid	−4.8	−3.8	5	3	5	3
Pyruvic acid	−4.5	−3.3	5	2	4	2
Shikimic acid	−5.3	−5.3	5	3	5	3
Succinic acid	−5.1	−4.0	3	2	3	2
Tartaric acid	−5.7	−5.5	7	4	7	4
Acarbose	−5.6	−5.4	7	4	6	4

Note: BA: Binding affinity, AG: α-glucosidase, AM: α-amylase, TIN: total number of intermolecular interactions, THB: total number of hydrogen bonds.

**Table 11:** MD values of hydroxycitric acid and acarbose complexes along with α-glucosidase.

MD Trajectory Values	Apo-Protein	Protein-Acarbose Complex	Protein- Hydroxycitric Acid Complex
RMSD (nm)	0.30–0.40	0.25–0.32	0.20–0.25
Rg (nm)	3.10–3.14	2.39–2.40	2.39–2.40
SASA (nm^2^)	350–370	240–250	240–250
Ligand H-bonds	-	7	8

**Table 12:** MD values of hydroxycitric acid and acarbose complexes along with α-amylase.

MD Trajectory Values	Apo-Protein	Protein-Acarbose Complex	Protein- Hydroxycitric Acid Complex
RMSD (nm)	0.20–0.30	0.25–0.35	0.20–0.25
Rg (nm)	2.31	2.31	2.31
SASA (nm^2^)	190–200	190–205	190–200
Ligand H-bonds	-	7	10

**Table 13:** Binding free energy values of target proteins complexed with hydroxycitric acid and acarbose.

Protein-Ligand Complexes	Types of Binding Free Energies				
	VDWE (kj/mol)	EE (kj/mol)	PSE (kj/mol)	SASAE (kj/mol)	BE (kj/mol)
AG-hydroxycitric acid	−220.118	−9.313	96.102	−28.166	−189.1022
AG-acarbose	−134.192	−4.813	62.125	−9.310	−90.102
AM-hydroxycitric acid	−218.568	−29.891	62.172	−21.886	−180.194
AM-acarbose	−130.161	−2.106	39.340	−9.564	−87.109

Note: VDWE: Van der Waal’s energy, EE: electrostatic energy, PSE: polar solvation energy, SASAE: solvent-accessible surface area energy, BE: binding energy.

## Data Availability

The datasets presented in this study can be found in online repositories. The names of the repository/repositories and accession number(s) can be found in the article.
